# Do international trade and investment agreements generate regulatory chill in public health policymaking? A case study of nutrition and alcohol policy in South Africa

**DOI:** 10.1186/s12992-021-00757-6

**Published:** 2021-09-06

**Authors:** Penelope Milsom, Richard Smith, Simon Moeketsi Modisenyane, Helen Walls

**Affiliations:** 1 Department of Global Health and Development, Faculty of Public Health and Policy, London School of Hygiene and Tropical Medicine, 15-17 Tavistock Place, Kings Cross, London, WC1H 9SH UK; 2 College of Medicine and Health, University of Exeter, Exeter, UK

**Keywords:** Policy chill, Regulatory chill, NCD prevention, Nutrition policy, Alcohol policy, Trade and investment agreements

## Abstract

**Background:**

Trade and health scholars have raised concern that international trade and particularly investment disputes may be used by transnational health harmful commodity corporations (THCCs) to effectively generate public health regulatory chill. The purpose of this study was to contribute to the limited evidence base of trade or investment dispute-related regulatory chill using a case study of nutrition and alcohol policy in South Africa.

**Methods:**

We conducted 35 semi-structured interviews with 36 key stakeholders involved in nutrition, alcohol and/or trade/investment policymaking in South Africa. Interview transcripts were analyzed using thematic analysis. We used Schram et al’s theory on three forms of regulatory chill (anticipatory, response and precedential) to guide the analysis. We report evidence on each form of regulatory chill as well as specific contextual factors that may influence the risk of regulatory chill.

**Results:**

Trade obligations were found to generate a significantly greater anticipatory-type chilling effect on nutrition and alcohol regulation than South Africa’s investment treaty obligations. Response chill was reported to have occurred in relation to South Africa’s proposed tobacco plain packaging regulation while awaiting the outcome of both Australia’s investor-state and WTO state-state disputes. No cases were reported of THCCs threatening an investor-state dispute over nutrition or food regulations, but there were reported cases of THCCs using arguments related to South Africa’s trade obligations to oppose policy action in these areas. No evidence of nutrition or alcohol policy precedential chill were identified. Factors affecting the risk of policy chill include legitimacy and perceived bias of the dispute system, costs involved in pursuing a regulation/defending a dispute and capacity to pay, social acceptability of the industry, a product’s perceived risk to health and confidence in a successful dispute outcome e.g. through cross-border policy learning.

**Conclusions:**

Our findings indicate that currently, South Africa’s trade obligations have a more prominent role in inhibiting nutrition and alcohol action than investment treaty-related concerns. However, given the potential for wider use of the ISDS mechanism by THCCs in the future, strategies to protect public health policy space in the context of both international trade and investment treaty and dispute settlement contexts remain important.

## Background

An equitable approach to addressing the growing burden of non-communicable diseases (NCDs) and their risk factors in low- and middle-income countries (LMICs) requires comprehensive population-level government interventions [1, 2]. Ultra-processed foods and hazardous alcohol use are two key areas for such regulation. Despite increased attention to these issues globally, political action to tackle food and alcohol environments has been limited. Such inaction can be described as policy ‘non-decision making’, encompassing deliberate decisions not to act, involuntary failures to act as well as unconscious inaction [3] by policymakers. As transnational ultra-processed food and alcohol companies (referred to as transnational health harmful commodity corporations or THCCs hereafter) increasingly turn their attention to LMIC markets for growth and profit [4–8], they are likely to intensify their efforts to promote and support non-decisions concerning nutrition and alcohol policy in these countries.

Various industry tactics to promote nutrition and alcohol policy non-decisions have been documented globally [7, 9–12]. However, the potential for THCCs to engage in ‘venue-shifting’, a strategy to claim alternative spaces of influence over policy decisions by shifting decision-making power from democratically-elected governments to other fora, including international trade and investment dispute settlement/arbitration venues, where their interests may be more likely to be prioritized [13, 14], has been relatively less well explored empirically. Although THCCs cannot themselves informally challenge or initiate a formal dispute at the World Trade Organization (WTO), they can encourage and support states to do so on their behalf [15]. For example, following significant lobbying by tobacco corporations, in 2012, five LMIC member-states initiated a WTO dispute against Australia for their proposed cigarette plain packaging legislation [16, 17]. States though, may be deterred from pursuing corporate interests in WTO fora due to concerns of retaliatory measures in other areas and norms of international diplomacy [18]. Even without state support, THCCs themselves have used international trade-related legal threats in an effort to generate a chilling effect on NCD prevention policy. For example, the alcohol industry threatened Thailand would face a WTO dispute if it adopted a proposed ban on alcohol advertising [19].

The investor-state dispute settlement mechanism (ISDS) however, now included in over 2000 active bilateral investment treaties (BITs) [20] as well as a number of important regional trade and investment agreements, provides a direct legal mechanism for corporations with narrow financial interests (and few disincentives to avoid spurious legal claims) to challenge domestic policies using international agreements [18]. ISDS allows THCCs to themselves bring claims for financial compensation against states in private international tribunals when they assess state action has compromised their investment [21]. To date, two of the most well-known investor-state disputes relevant to public health have been the cases of Philip Morris Asia vs Australia and Phillip Morris International vs Uruguay for their tobacco plain packaging and graphic warning labelling policies, respectively [22, 23]. In both cases arbitrators ruled in favour of the state, although in Australia this was on jurisdictional, not substantive grounds. Notably, given the lack of precedent in investment arbitration, tobacco companies may continue to threaten or pursue investment arbitration for similar regulations elsewhere [24].

Trade and health scholars have raised concern that through active or threatened venue-shifting to investor-state or WTO state-state dispute settlement, THCCs may be effective in generating public health ‘regulatory chill’ [25, 26], a specific kind of policy non-decision, where a government delays, compromises, or abandons the formulation or adoption of bone fide regulation in the public interest to avoid a trade or investment dispute [25, 27, 28]. Governments are incentivised to avoid a trade dispute due to the substantial legal, administrative and economic costs and the possible impact on future trade negotiations [28, 29]. However, a real or perceived threat of an investor-state dispute may be an especially powerful driver of public health policy chill [18, 30] as compared to a potential WTO dispute given the particularly high financial cost of engaging in an investment dispute [31]; potential award of financial compensation to investors [32, 33]; vague and expansive nature of foreign investment protection provisions; unpredictability of a dispute’s outcome; lack of appeal mechanism and potential arbitrator conflict of interest. With limited financial and technical resources and potentially also economic dependency on trade and investment from wealthier countries, LMICs may be especially vulnerable to regulatory chill [34].

In this paper, we explore and test three distinct forms of trade or investment dispute-related regulatory chill, similar to those described by Schram et al. (2018). *Specific response chill* refers to a chilling effect on a specific proposed/adopted measure after a government becomes aware of the threat of a potential dispute in relation to such a regulation [25] which may be due to a dispute being pursued in another country. *Anticipatory chill* occurs in situations where policymakers take into account potential disputes during the policy development process, hampering regulatory progress across a range of public health policy areas [25]. Lastly, *precedential chill* is where policymakers change or abandon a particular regulation in response to a settled or resolved trade or investment dispute due to concern of future disputes based on the same regulation [25].

On reviewing the literature, we identified very few empirical case studies broadly investigating investment dispute-related regulatory chill, particularly in LMICs. A Canadian case study by Côte (2014) including health and safety and environmental regulators found little evidence of investment dispute-related regulatory chill [35]. Côte also conducted in-depth interviews and surveys with tobacco control regulators from 11 and 28 countries respectively, with similar findings. A separate, 2016 Canadian study by Van Harten and Scott, including interviews with officials in ministries with an environmental or trade mandate in Ontario, concluded that the Ministry of Health had changed its policy-making process to account for the risk of a trade or investment dispute including via adopting regulatory impact assessments and legal vetting procedures [36]. A few studies have also reported on specific cases of response chill. For example, Curran and Eckhardt (2017) reported how industry threats of investment arbitration using NAFTA’s Investment Chapter 11 heavily impacted the Canadian government’s decision to abandon their proposed tobacco plain-packaging regulation in the 1990s [29]. Notably however, around two decades later, analyses explore how Australia and Uruguay avoided a chilling effect on tobacco control legislation despite threats and eventual pursuit of investment arbitration by tobacco corporations [16, 37].

Analysis of regulatory chill resulting from government efforts to avoid a trade dispute is also relatively limited. Some have shed light on why, despite eventual escalation to formal WTO disputes, the governments of Australia and Uruguay continued to pursue novel tobacco control measures [38–40]. Other studies have focused on earlier stages in the WTO complaint process by analyzing trade challenges raised within the WTO’s Technical Barriers to Trade (TBT) Committee meetings, to assess whether WTO rules have been invoked in an effort to challenge proposed health regulations [13, 41–44]. The largest of these include two studies by Barlow and colleagues identifying that of all the challenges or trade concerns raised by members at the WTO’s TBT Committee between 1995 and 2016, 250 concerned regulations aimed at protecting human health or safety [45], and of these 93 were over food, beverage and tobacco regulations [28]. However, very limited empirical analysis has been conducted to identify the extent to which such informal trade challenges or formal WTO disputes generate a chilling effect on NCD prevention policy action. The few studies we identified include for example, Barlow and colleagues’ (2018) that reported four case studies where informal trade challenges within the WTO TBT Committee were associated with food or beverage policy change or delay [28].

The purpose of this study therefore is to contribute to the particularly limited evidence base for trade or investment dispute-related regulatory chill using an in-depth case study analysis of nutrition and alcohol policy non-decisions in the LMIC country context of South Africa. The aim was to understand to what extent trade or investment agreements/rules are used by industry or potentially also economic policy actors as a tool to promote nutrition and alcohol policy non-decisions; to what extent, why and how the threat of an investor-state dispute as compared to a state-state WTO dispute contributes to public health regulatory chill; which types of regulatory chill may be occurring; and to identify any contextual factors, particularly relevant for LMICs, that may be either protective or increase vulnerability to regulatory chill.

Ethical approval for this work was obtained from the London School of Hygiene and Tropical Medicine (28 August 2018) and the University of Cape Town (12 December 2018).

## Methods

### Case study selection

South Africa, a middle-income country, was selected as an appropriate single case study for a number of reasons including its relatively high engagement in trade and investment agreements, previous exposure to trade and investment disputes and stated commitment to addressing the rising burden of NCDs. These are outlined in detail below as well as why they contributed to the selection of South Africa as the case study for this work.

#### Trade and investment context

At the time of this study, South Africa was engaged in a number of trade and investment agreements, exposing it to threats of both WTO and ISDS arbitration. After Apartheid ended in 1994, South Africa rapidly entered into a number of trade and investment agreements in order to access foreign markets for South African goods and promote foreign direct investment into the country. In 1995 it became a member of the WTO, signed a Free Trade Area with the Southern African Development Community (SADC) in 1996, a further 22 bilateral investment agreements between 1997 and 2003 and a bilateral trade agreement with the European Union (EU) in 1999 [46].

Within this context, South Africa has been subject to both trade and investment disputes. Since 1995 the country has been the respondent in five WTO disputes [47]. Additionally in 1997, forty pharmaceutical companies brought a domestic legal challenge against South Africa claiming the country’s 1997 Medicines and Related Substances Control Amendment Act that allowed the use of parallel importation and compulsory licensing of affordable generic medicines, was in violation of the WTO’s Agreement on Trade-Related Aspects of Intellectual Property Rights (TRIPS) [48].

The risk of investment arbitration has also been within the political consciousness in South Africa given the recent ISDS cases against Australia and Uruguay for proposed tobacco control regulations and South Africa’s own previous exposure to two investment disputes which (along with other cases globally) prompted a review of all South Africa’s BITs in 2010. The review concluded that South Africa’s ‘first generation’ BITs contained significant ambiguity in the core legal provisions protecting investor rights and potentially opened the door for narrow foreign commercial interests to challenge legitimate, constitutional, democratic public policy in unpredictable international investor-state arbitration [49]. Based on the review’s recommendations, the South African government terminated a number of existing BITs and instead sought to provide sufficient investment protection through a new Protection of Investment Act that came into effect in 2018 that confirmed South Africa’s commitment to an open, transparent environment for foreign investment, attempted to secure a balance of rights and obligations for all investors and reaffirmed the government’s right to regulate in the public interest [50].

Together with the other SADC countries, South Africa has also participated in a new model BIT [49] that allows South Africa to opt-out of ISDS in any future BITs, requires investors to exhaust local remedies before proceeding to arbitration, and provides the basis for government counterclaims and legal action against investors for treaty breaches [51]. Additionally in 2019, South Africa made a submission to UNCITRAL discussing a range of possible reforms to the ISDS system including to protect domestic policy space [52]. However, at the time of this research, while South Africa has terminated 12 BITs, it remained subject to potential investor-state arbitration under 12 ongoing BITs to which it was party, and under ‘survival’ clauses of terminated agreements.

#### Nutrition and alcohol-related NCD prevention policy context

Given that NCDs now account for 51% of all deaths annually [53] and alcohol-related harm remains persistently high in South Africa [54], healthy diets and reducing harmful alcohol consumption have become key public health priorities. These are reflected in the Strategy for the Prevention and Control of Obesity 2015–2020 and Strategic Plan for the Prevention and Control of NCDs 2013–17 [55, 56]. Although the South African government has adopted a number of internationally recommended policies for the prevention and control of NCDs in the areas of nutrition and alcohol [57, 58], there are a number of policies/regulations that have been proposed but significantly delayed, drafted but not progressed, or adopted but after significant delay and/or re-formulated such that their effectiveness is reduced. These policy non-decisions (outlined in Table 1) were discussed during stakeholder interviews to explore whether the risk of a trade and/or investment dispute had influenced the policy process.

**Table 1:** Overview of alcohol and diet-related NCD prevention policy non-decisions in South Africa

Description of policy or regulation	Status
Ban on marketing of unhealthy food and non-alcoholic beverages to all school-aged children	Drafted in 2014, not progressed.
Mandatory front-of-pack nutrition labelling of food and non-alcoholic beverages	Drafted in 2014, remains under development.
Tax on sugar sweetened beverages	Introduced in 2019 at 11%, reduced from the originally proposed 20% (supported by evidence indicating this higher rate would be more effective).
Ban on marketing of infant formula	Comprehensive policy adopted in 2012 but policy process significantly delayed.
Ban on marketing of alcoholic beverages	Currently under the Liquor Act of 2003 it is prohibited to advertise alcohol targeting minors or to use false or misleading advertising [59]. A new Control of Marketing of Alcoholic Beverages Bill drafted in 2013, includes provisions to ban advertising, sports sponsorships and promotion of alcoholic beverages [60] has not progressed.
Health warning labelling on alcoholic beverage containers	In 2017 draft amendments to existing health warning labelling regulation (2007) were published, increasing the size of warning labels and requiring regular rotation of seven heath warning messages [61]. These ammendments were later repealed in 2020.
Increasing the drinking age to 21, banning alcohol trade within 100 m of schools and churches and liability clauses for alcohol retailers.	The draft Liquor amendment Bill of 2016 [62] containing these (among other) regulations has undergone three revisions and remains under consideration.
Controlling the production and sale of certain alcoholic products by changing the alcohol content of what was deemed as liquor from 1% of volume to 0.5% and to regulate the import and export of certain alcoholic products.	The draft Liquor Products Amendment Bill 2016 [63] containing these (among other) regulations has undergone three revisions and remains under consideration.

Additionally, given its geographical position, infrastructure and relatively open economy, South Africa is a strategic hub from which THCCs can develop new markets across Africa [7]. This combined with South Africa’s status as a regional policy leader, may mean THCCs have a particular interest in securing and maintaining a favourable regulatory environment in South Africa to prevent regional and continental policy transfer [59].

In summary, South Africa was selected as a useful case study given the country’s exposure to international trade and investment rules and dispute systems; political awareness of trade and investment obligations and dispute risk; recognition of unhealthy diets and alcohol-related harm as public health problems requiring policy action; and significant multinational food and alcohol corporate presence in South Africa. These characteristics allowed for analysis of if and how trade and/or investment rules/disputes threats may be used by external actors, particularly THCCs, to effectively generate NCD prevention regulatory chill.

### Data collection

We developed a semi-structured interview guide containing questions to elicit key actors’ understanding and experience of how South Africa’s international trade and investment obligations might influence nutrition and alcohol harm reduction policy processes; and the strategic approaches adopted by different stakeholders to achieve their desired trade/health objectives. The interview guide was piloted with local experts within academia and government and adapted accordingly before use. An initial stakeholder mapping was also undertaken to identify key participants in the Department of Health (DoH), Department of Trade and Industry (DTI), Department of Agriculture Forestry and Fisheries (DAFF) as well as relevant international organisations (IGOs)/non-government organisations (NGOs)/civil society organisations (CSOs), industry and academia with experience or expert knowledge on nutrition and alcohol policy issues with potential relevance to international trade/investment; or trade and investment policy development and negotiations. Key stakeholders identified in the mapping process were then invited to participate in an interview. Subsequently, snow-ball sampling resulted in additional stakeholders from within the Department of Social Development (DSD); National Treasury and current and former Health Attachés for South African Permanent Mission to the United Nations Office in Geneva or South African Embassy in Washington DC being invited to participate. In total 74 stakeholders were contacted and invited to take part in in a one-hour semi-structured interview. Thirty-six agreed, 23 did not respond and 12 declined to be interviewed (Table 2). While we attempted to recruit government stakeholders in both senior technical and more political roles, it was very challenging to recruit the latter.

**Table 2:** Summary of study participants

Stakeholder group	Key stakeholder invited to interview	Key stakeholder included in the analysis			
		Nutrition	Alcohol	Cross- Cutting*	Total interviewed
Department of Health	17	7	1	3	10
Department. of Trade and Industry	14	0	2	6	7
National Treasury	4	1	0	1	2
Department of Agriculture, Forestry and Fisheries	6	2	0	0	2
Department of Social Development	1	0	1	0	1
Intergovernmental organisations, non-government organisations and civil society organisations	8	4	2	0	6
Multinational food and alcohol corporations (originating both from within and outside South Africa)	10	2	2	0	3
Academics	11	3	2	0	5
Health Attachés for South African Embassy in Geneva or Washington DC (current or past)	6	0	0	0	0
**Total**	**77**	**19**	**10**	**10**	**36**

* The Cross-cutting category refers to stakeholders with experience or knowledge relevant to both nutrition and alcohol policy

(incidentally a number of them also have experience/knowledge relating to tobacco control)

Between April 2019 and February 2020, 35 interviews were conducted with 36 participants either in-person in Cape Town/Pretoria or via phone/teleconference. Written consent was obtained for all interviews. All government participants were chief or deputy directors within their respective departments with one deputy director general. In this work, government stakeholders directly involved in either agenda-setting or policy formulation are referred to as ‘policymakers’, while stakeholders in more political roles are referred to as government 'officials'. Industry representatives were governance and regulatory experts, IGO, NGO and CSO representatives had each been engaged in recent relevant nutrition or alcohol policy processes in South Africa. Where a stakeholder did not given permission to identify their institutional affiliation, they are simply referred to as a trade, health, or industry ‘stakeholder’.

A small number of DoH stakeholders interviewed were in higher-level positions overseeing, or with broad knowledge of, multiple NCD policy areas and had an understanding of the risk of trade or investment disputes in relation to tobacco control. A number of trade policymakers also had similar knowledge. As such, while the focus of the research and interviews was on nutrition and alcohol policy, tobacco control issues were also raised by some interviewees and reported in the research findings.

Thirty-three out of the 35 interviews were recorded. Detailed notes were taken during the two unrecorded interviews. All recorded interviews were later transcribed in full. After each interview, the audio recordings or notes were reviewed to inform necessary adaptations to the interview guide and to identify the need for further interviews.

### Analysis

We analysed the data using thematic content analysis. Codes were initially developed deductively, based on the three forms of regulatory chill outlined previously. Additional codes were developed inductively during the analysis. Coding was conducted in NVivo (version 12.6.0) to ensure consistency and transparency in the coding process. Coded extracts were then imported into Word documents organized according to main themes to identify patterns across key informant interviews.

## Results

Results are reported for each form of regulatory chill (anticipatory, response and precedential) and comparisons are drawn between any identified chilling effect generated by a perceived risk of an investor-state versus a WTO state-state dispute. We also report identified concessions on public health regulations made during trade negotiations. Finally, conditions that may increase the risk of, or protect against, regulatory chill are described.

### Anticipatory chill

#### Limited consideration of investor-state dispute risk during nutrition and alcohol policymaking

While health policymakers in higher-level positions (and with a broad understanding across NCD policy areas) were aware of the specific risk of an investor-state dispute in relation to tobacco control (given the recent ISDS cases brought against Australia and Uruguay), this awareness or perceived risk had generally not spilled-over into nutrition and alcohol policymaking spaces. Most policymakers exclusively involved in nutrition or alcohol policymaking within the DoH were not specifically aware of the risk of investor-state disputes and did not differentiate between obligations within trade agreements and BITs or different legal fora – WTO, international investment arbitration or domestic litigation. Although aware of South Africa’s international investment obligations, one technical officer within the DoH commented:

*“It* [the threat of an investment dispute] *is not something that we have considered I must say, so it is difficult to comment on. I am aware of the tobacco issues. But in this case of alcohol, not at all. It’s not something that has been on the table.” *[DHA1].

Nutrition and alcohol advocates within CSOs and NGOs broadly lacked awareness of South Africa’s international investment obligations and exposure to potential investor-state disputes.

However, various trade and health policymakers confirmed that all public health regulations were vetted by state legal advisors to ensure compliance with South Africa’s constitutional and international legal obligations, including under existing trade agreements and BITs. One trade official commented, for example:

*“there is an enormous amount of resources that go into this … to say what are the trade effects? And then you’ve got to make a judgment in terms of the agreements to say yes you can do it for these reasons, but you have to do it in the way that least restricts trade”* [DTI3].

A nutrition policymaker within the DoH also stated:

*“we make sure that whenever we come up with legislation, our lawyers will get that, and should there be any sign of any possible disputes, they would have to advise that this might impact in terms of trade* [or investment obligations]*.”* [DHN1].

This suggests that despite limited awareness amongst most but not all nutrition and alcohol policymakers, there was some cursory awareness of investment-related risk assessment being internalized in the policy development process with the potential to generate a degree of anticipatory chill.

#### Internalization of trade dispute risk in nutrition and alcohol policymaking

In contrast to limited awareness of international investment obligations and risk of ISDS, health policymakers were generally aware of the risk of generating ‘trade concerns’ from trading partners and industry or potential escalation to a formal WTO dispute if health policy was not compliant with South Africa’s trade obligations. Policymakers described that compliance with WTO rules had contributed to the internalization of a number of principles during policymaking, particularly for trade-sensitive regulations (e.g. nutrition and alcohol health warning labelling). These included revising the regulation to ensure it is as least trade restrictive as possible, adopting a strict evidence-based approach to policymaking and when local evidence was not available, ensuring policies aligned with international standards or guidelines. Following these principles were considered by trade actors not to restrict regulatory space for nutrition and alcohol harm reduction. One trade policymaker explained, for example:

*“If the DoH identifies the need for some kind of you know labelling … it will be done because it’s been identified as a need and then that will be a scientifically grounded decision … they will ask us what the implications are for trade and they will make sure that the way that it’s carried out in a manner consistent with our obligations. And if we are clear that its consistent with our obligations … that it’s evidence-based … that it will be applied to deal with the particular health problem then we will be able to convince our principal and proceed”* [DTI02].

However, internalizing these principles in health policy processes to comply with trade rules was reported by health policymakers to limit the scope of policies and policy design options available; delay the policy process; and was burdensome on limited DoH resources. As such, trade obligations generated a significantly greater anticipatory-type chilling effect on nutrition and alcohol regulation than South Africa’s investment obligations. One DoH policymaker remarked for example:

“...*we’ve now got to work harder in terms of how we’re then going to defend, how we approach this because whatever we put on the label, it can’t hinder any trade*.” [DHN2].

Alcohol and food labelling were particularly recognised as potential technical barriers to trade and ensuring these and any other trade-sensitive health regulations were as least trade restrictive as possible was internalized in policy development. Unacceptably high costs of implementing a regulation for THCCs importing into South Africa were particularly mentioned as a technical barrier to trade. As such, minimizing the cost to importers of a heath regulation during policy development was considered important, particularly by trade policy actors and was a potential driver of policy non-decisions. For example, as one trade policymaker commented when asked whether nutrition labelling would be considered a technical barrier to trade:

*“… it also depends on what the manufacturers, the cost for them will be, and for trading partners and the manufacturers in other countries from where we import, what their views are”* [DTI1].

Trade obligations and concern to avoid triggering informal trade challenges that may escalate to formal disputes have contributed to the internalization of a strict ‘evidence-based’ approach to policymaking and was identified as a key driver of anticipatory chill. Evidence of the need for regulation (e.g. obesity or fetal alcohol syndrome prevalence) and usually also of likely policy effectiveness, including specifically in the South African context, was considered necessary which caused delays, especially given limited DoH research funding. As one DoH policymaker reflected:

“*it’s delaying it* [front-of-pack nutrition labelling policy process] *to the extent that those who've advocated for this policy are saying ‘but you’re taking too long’ … but we have to put in place the scientific evidence and all the consumer acceptance … so that it can be defended if it does come up as a trade dispute”* [DHN2].

Another health stakeholder explained, making reference to the Specific Trade Concerns raised six times by member states at the WTO TBT Committee in relation to Thailand’s proposed front-of-pack traffic-light nutrition label and 'children should take less' warning on snack foods proposed in 2006 [60]:

*“some of these international organisations they will say that there is no robust evidence on the issue of food labelling legislation that we are proposing and if you go ahead with that food labelling legislation then, like in Thailand, you will be subjected to WTO agreements and then you go through the WTO dispute resolution mechanism”* [H1].

Notably, Thailand’s nutrition labelling regulation was not adopted until 2013 and with modifications that potentially compromised its effectiveness [60].

In relation to South Africa’s proposed tobacco plain packaging regulation, another health stakeholder commented:

“*when they* [industry] *are threatening, you also want to make sure that you have enough evidence that could stand in a court of law. So, for all those areas that they started threatening we were able to go and search for more in-depth and more convincing evidence so that by the time they take us to court, we are ready because they are already indicating that they will take us to court”* [H2].

While local evidence to support the need for and likely effectiveness of a regulation was considered grounds to safely diverge from international standards/guidelines, limited DoH funding for research meant health policymakers were often forced to rely on international standards/guidelines to determine the policy agenda in order to avoid trade challenges. As one DoH policymaker commented:

*“for us as a developing country, we don’t have the resources to go about doing the science, so we often have to rely on donors, international donors that can assist us to develop this science whereas if it’s already in Codex or it’s already in WHO, when it comes from a health policy perspective, we can then say well, the policy narrative comes from the WHO, therefore it’s something that we need to look at.*” [DHN3].

Without local research, the additional lack of guidance from Codex on front-of-pack nutrition labelling had also contribute to delayed progress on nutrition labelling.

Trade policymakers also commented on the importance of adhering to international standards but that deviation from these standards was acceptable if adequately robust evidence existed to support an alternative measure. One DTI policymaker reported:

*“where there is an international standard in place you must use that as a guide, and where there are situations in your country where the international standard won’t address your objective for the regulation, you can deviate from the international standard, but that should be evidence-based.”* [DTI1].

A number of stakeholders mentioned that the obligation to notify WTO of any proposed regulation provided foreign corporations with another channel, either directly or through their home governments, to lobby and prolong the policy process. However, this process was broadly considered necessary and important to ensure transparency and predictability in the policy environment despite being time and resource intensive. Health policymakers also considered that spending sufficient time consulting with international stakeholders was important to prevent THCCs taking legal action, as explained by one DoH policymaker:

*“The legislation* [nutrition labelling regulation] *is still in the consultation phase… we didn’t want to rush in in terms of bringing in this legislation because we know the impact it’s going to have … we didn’t want to … be taken to court* [by a company] *saying that they were never consulted. We wanted to avoid it. Hence, even our international counterparts, we sent it out to them and said this is what South Africa’s going to come up with – do you have any comments?”* [DHN1].

### Response chill

Trade officials reported that the South African government had delayed progress on their proposed tobacco plain packaging regulation by about two years until the outcome of both Australia’s ISDS and WTO cases were known, suggesting a degree of response chill had occurred in the area of tobacco control. Adopting a ‘wait and see’ approach was based on a reluctance to expend resources on developing and implementing a regulation they would later have to reverse if the same regulation in another country was judged in arbitration to be in violation of either international investment or WTO rules. As one trade official explained:

*“if you were watching a case under a bilateral investment treaty, and you went ahead and implemented that same regulation and the case was found in favour of the investor, then you could just see them lining up in South Africa to proceed in the same way, so you’d have that* [chilling] *effect but at the WTO the fact that this case* [involving Australia’s tobacco plain packaging regulation] *was going on, we didn’t know what the outcome would be, so would you go ahead and implement it only to have to reverse it afterwards because the award went against the Australians? So yeah so it would have the same* [chilling] *effect.”* [DTI3].

#### Use of investment protection rules or threat of investor-state dispute as a corporate tactic to generate nutrition or alchol regulatory chill

There were no definitive cases reported by key informants of THCCs threatening to initiate an investor-state dispute in an effort to generate a chilling effect on a specific nutrition or alcohol regulation in South Africa. Notably, one alcohol industry representative denied that they were even aware of the ISDS mechanism. Further, senior trade officials within the DTI reflected that from their perspective, avoiding a WTO dispute was of equal concern as avoiding an investor-state dispute, particularly due to the very high perceived costs involved with both.

Civil society representatives and academics perceived that resorting to the use of ‘hard’ legal tactics has to date been generally unnecessary for food and alcohol corporations. Instead, they are considered legitimate stakeholders in the policymaking process and can effectively apply ‘softer’ mechanism of power to expand access to and influence within policymaking spaces. One academic reflected for example:

*“… they* [THCCs] *probably haven’t needed to do that* [use investor-state disputes] *because they’re using other approaches like embedding themselves with senior government officials”* [RA2].

Another academic shared a similar view:

“*they’re so close* [government and the alcohol industry] *that they don’t need to bring in these threats of international trade agreements because they’ve got enough power within the country to push policymakers.”* [RA1].

While a number of CSO representatives and academics were concerned that the alcohol and food industries would, if necessary, use investment arbitration in the future, preserving an amicable relationship with government was considered a key motivation for industry to avoid, wherever possible, adopting such ‘hard’ tactics of influence. For example, one health official commented:

*“I think they try not to offend government* [with legal threats] *and sometimes government doesn’t respond well to threats. Sometimes they have their mind more on convincing- to say look, if this goes ahead we’re going to have to scale down our factory, and people are going to lose jobs because our sale of sugar is going to decline”* [TS1].

These views were supported by one alcohol industry representative in relation to the proposed alcohol health warning labelling which they argued was ambiguous and contrary to other domestic law:

*“*[in relation to the] *health warning regulations we had to make a decision whether we would take the DoH to court. And it was an incredibly difficult decision because … they are regulators and you might win that battle but lose the war ultimately … the decision that we took at the time is:let’s continue finding ways to find some solutions with the DoH but use our courts as a last resort”.* [AI1].

#### Use of trade rules/informal trade challenges to generate a chilling effect on nutrition and alcohol policy

Respondents described a number of cases in which South Africa’s trade obligations were used by either trading partners or THCCs to generate a chilling effect on specific nutrition or alcohol regulations. The number of examples described may well be underestimated since it was acknowledged by some health policymakers that pressure from trading partners for South Africa to abandon certain regulations potentially occurred between high-level political actors within closed informal political spaces.

Trading partners and THCCs had raised ‘trade concerns’ and/or sought bilateral consultation in relation to South Africa’s proposed front-of-pack nutrition labelling of processed foods. For example, one DoH policymaker reported having resisted attempts by other countries to pressure South Africa into aligning their food labelling regulations with other countries to minimize costs to their companies importing into South Africa and to avoid generating an unnecessary trade barrier.

While health policymakers denied that other countries’ proposed nutrition labelling being raised as a ‘specific trade concern’ within the WTO’s TBT Committee had delayed progress on South Africa’s own labelling regulation, policymakers were assessing these cases as part of their policy development process and proceeding cautiously.

In relation to the tax on sugar-sweetened beverages introduced in 2018, health policymakers reported that a sugar-producing European country had attempted to pressure South Africa into dropping the regulation claiming that it would affect global sugar production and was in violation of South Africa’s trade commitments. However, one health actor commented "*in the end the trade side was also overridden by the health"* [H3] and the tax was introduced, although at just 11%, not the originally proposed 20%.

It was also reported by nutrition policymakers that industry had argued the originally proposed Regulations Relating to Foodstuffs for Infants and Young Children banning marketing of breastmilk substitutes (eventually introduced in 2012), would create unnecessary barriers to trade; that certain elements went beyond what was recommended by Codex and the WHO’s International Code of Marketing of Breastmilk Substitutes (e.g. including pacifiers/dummies); or did not have sufficient supporting evidence (e.g. banning marketing of complimentary foods). However, these threats did not dissuade the DoH from adopting one of the most comprehensive set of regulations relating to the marketing of infant formula globally and in line with WHO guidelines.

In 2014 the DoH proposed amendments to their Regulations Relating to Health Messages on Container Labels of Alcoholic Beverages, increasing the size of the warnings to one-eighth of the container and rotating each of the seven warnings within every twelve-month period. After notifying the WTO of the amendment, the regulation was challenged informally at the TBT Committee by the EU and Canada over concerns it would create barriers to trade for small and medium producers [61]. Subsequently, the local alcohol industry as well as trading partners (including the EU and US) and foreign transnational alcohol corporations have bilaterally engaged the DoH raising concerns about ambiguity of the regulation; problem with the wording of the health messages, accepting for example ‘don’t drink and drive’ but not ‘alcohol may be a danger to your health’; impracticality/technical feasibility of the proposed size of the labels; the cost to manufacturers of such frequent rotation of messages; and lack of sufficient evidence of the regulation’s effectiveness in reducing alcohol-related harm. It was mentioned by a few health policymakers that transnational alcohol companies had complained that, for a number of the reasons outlined, the labelling requirements would create unnecessary barriers to trade.

Ultimately however, despite a reported earlier consensus between the DoH and DTI in favour of amending the alcohol health warning labelling regulation, in October 2020, the DoH repealed the proposed amendments due to the challenges of implementing the regulation raised by local industry (e.g., the difficulty in calculating one-eight of the surface area on an alcohol container) and potentially also international stakeholders' concern that the regulation created unnecessary barriers to trade. The DoH planned to review the regulation in light of informal discussions with the WHO and their discussion paper on policy options for alcohol labelling. This process indicates the requirement for very specific international guidance on the design, size and content of health warning labels based on scientific evidence.

When asked more generally about the use of trade rules as a strategy to influence South Africa’s regulatory environment, one alcohol industry representative reflected:

*“...it’s a long, drawn-out process, even as a business. We would never go to a government and say that this government is in contravention of the WTO, without being a hundred percent certain.”* [AI2].

Another foreign transnational alcohol corporation representative commented that while “*using international trade rules to limit policy”* [IA2] had been considered by the alcohol industry, it was in fact very difficult to achieve.

There was however indication that the alcohol industry attempted to enlist the South African government to act on their behalf at the WTO in an effort to chill policy progress in South Africa’s trading partner countries. This was explained by a trade official:

*“Industries will come and they’ll make a case and they’ll go through NEDLAC and they’ll write to the ministers, they’ll write to the president, they’ll speak to all of the officials and they try to make their case* [for filing a WTO complaint against a trading partner] *and then you’d have to make an assessment of whether or not the case is legitimate, whether or not you have a chance of winning the case.”* [DTI3].

An alcohol industry representative commented however, that their industry did not contribute enough to GDP to be in a position to convince the SA government to act on their behalf within WTO fora. Instead the alcohol industry was able to utilise its global business network through, for example the World Wine Trade Group that includes wine producers and distributers in the US, Canada, Chile, Australia, New Zealand, Uruguay, Argentina and Georgia. For example, in relation to Scotland’s proposed minimum unit pricing regulation an alcohol industry representative commented:

“*we asked our counterparts in those countries to please speak to their governments. And again, you speak to the ones who are most likely to help. And you know the US government was willing to listen to its industry and raise concerns*”.

Together these findings again indicate that South Africa’s trade obligations are currently a much more relevant tool of influence to promote nutrition and alcohol policy non-decisions than any problem of response chill resulting from threats of investment arbitration.

### Precedential chill

Given that no previous WTO or investor-state disputes have been in relation to a specific nutrition or alcohol harm reduction regulation in South Africa or elsewhere, no cases of precedential chill in these policy areas were identified. However again, trade officials reported South African government’s confidence to proceed with their tobacco plain packaging proposal was significantly boosted by the positive outcomes in Australia’s investor-state dispute despite the lack of precedent in international investment case law. This suggests that had the opposite outcome been reached in this case, precedential chill may have occurred for tobacco plain packaging in South Africa.

The outcome of the WTO dispute against Australia was however reported by both trade and health actors to have equally influenced South Africa’s decision to proceed with implementing their own regulation. One trade official explained this was due to an understanding that once precedents are established in WTO case law, they usually hold in future cases. Therefore it may have been considered too risky for South Africa to proceed with plain packaging if Australia has lost their case:

*“if it had gone against Australia perhaps there would have been a re-evaluation* [of the policy in South Africa] *and to then take into account the risks of another challenge to us and you know once the precedent is set, then it’s very difficult to win the case after that, so the risk of being challenged successfully would have gone up and so … we would have to make an assessment whether not it was worth taking that risk*.” [DTI3].

### Concessions on public health regulations during trade negotiations

In addition to concerns of post-agreement trade rule violations, trade policymakers described the potential for health policy non-decisions to be promoted during trade agreement negotiations.

There was concern amongst high-level trade officials that international trade rules, particularly those outside the WTO systems, so-called ‘WTO-plus’ or ‘WTO-extra’ commitments, had the potential to restrict domestic public policy space for addressing development challenges. As such South Africa generally tried to negotiate agreements within the WTO framework. As one trade official explained:

*“we still sit with huge unemployment and rural under-development... So you want to address both issues, you don’t want to be tied up in agreements that prevent you from doing certain actions that are in the public’s interest.*” [DTI3].

However, it was fairly widely perceived that, as a developing country, South Africa was often required to make concessions during trade negotiations with larger more powerful economies, including further opening their markets for processed foods products and alcohol. As one alcohol industry representative stated:

“*they* [the DTI] *don’t start looking at alcohol policy and say well you know, should we be allowing alcohol to come in duty free? It’s the powers of negotiators at a trade block level that will determine the outcome*” [AI1].

In 2015 the US was reported to have threatened to cut access of approximately 6000 South African products, including wine, to the US market under the African Growth and Opportunity Act (AGOA) Agreement if South Africa did not lift an anti-dumping duty and other trade barriers to imported US chicken products. Ultimately, South Africa agreed to reduce barriers to US chicken imports which also required relaxing poultry food and safety standards that some suggested had potential direct public health impacts. Others mentioned the indirect public health impacts relating to the devastating economic impact on local poultry farmers who could not compete with the high volume of cheap imported US chicken cuts. There was however a general perception that, ultimately, the economic benefits outweighed the health impacts, as one health stakeholder explained:

“[we] *looked at the cost and benefits ultimately and we then realised that, in the long run, it will be in our best interest to relax some of the health and safety regulations for a bigger agenda or for a bigger good*.” [H1].

### Conditions that influence regulatory chill or trade-related policy non-decisions

Both trade and health policymakers discussed various conditions that may directly or indirectly increase the likelihood of, or protect against trade or investment dispute-related regulatory chill.

#### Perceptions of trade and investment rules and dispute settlement systems

The first set of conditions relates to perceptions of the international trade and investment rules and dispute settlement systems themselves, however these were primarily discussed in general terms, not in relation to specific cases of nutrition or alcohol regulatory chill. While some trade policymakers were confident that existing safeguards within the Sanitary and Phytosanitary Agreement (SPS) and TBT Agreement provided sufficient protection for nutrition and alcohol harm reduction regulation, another high-level trade official was concerned that WTO agreements “*were not entirely balanced”* and tended to prioritize trade over health objectives. However, this comment was made in relation to TRIPS and access to medicines, not nutrition or alcohol regulation. The same trade official also reflected that the WTO dispute settlement system was structured in a way that prioritized trade over health:

*“it’s quite tenuous in a sense that these serious health considerations would be subject to a decision by panellists that have in their mind the trade implications overwhelmingly... you get a chance of a bias in the WTO towards trade … they’re highly competent people but this* [health] *is not their field.”* [DTI3].

However, another trade policymaker felt that over time, WTO norms had shifted such that expert input from the WHO was increasingly sought and considered during health-relevant arbitration.

Generally though, the WTO as opposed to the investor-state dispute system was still perceived as a preferred option, partly since it had been agreed on by all WTO member states and provides a buffer against weak claims by industry:

*“the WTO mechanism is seen to be a better option, it’s also not … private companies that challenge governments, its* [other] *states. They* [private corporations] *have to convince their government to take up the challenge in order to launch it … so there’s an advantage”* [DTI3].

Trade policymakers recognized a number of characteristics of the ISDS system which may increase the risk of regulatory chill which, not surprisingly, aligned with the findings of the 2010 Review of South Africa’s BITs. These included a lack of perceived legitimacy of the ISDS process since cases are brought by private corporations against a government and the outcome decided by three private arbitrators; a lack of precedent and consistency in arbitral decisions; conflict of interest of arbitrators and lawyers; and cost of arbitration itself as well as potential investor compensation.

While in theory these concerns meant the threat of an investor-state dispute may generate greater uncertainty and concern than the threat of a WTO dispute, in practice, the perceived high costs associated with either could potentially have a chilling effect on health policy. One trade official reported for example:

*“ … the costs become a really important consideration. And the longer they go on the more costly that becomes and many developing countries simply don’t have the finances to pursue these cases … even when they would want to … and may accede to the demands of the claimants more easily than a developed country that … is prepared to fight the case with the best available lawyers over a period of time”* [DTI3].

The cost of trade sanctions imposed by a trading partner in response to an identified or perceived violation of South Africa’s trade obligations was also noted to be a major consideration.

#### Country-related characteristics

Stakeholders within the DTI also identified a number of country-related characteristics, largely determined by a state’s level of economic development, that may increase the likelihood of regulatory chill in South Africa and other LMIC countries. Limited institutional capacity for analysing trade and investment treaty texts (as had previously occurred in South Africa) and their ongoing status as primarily a ‘rule-taker’ in treaty negotiations with larger economies were considered by trade policymakers to potentially make it difficult to protect policy space to regulate in the public interest. Lack of technical capacity and human resources was also reported to make it challenging for South Africa to engage in trade negotiations or, monitor and assess new regulations and procedures within the multiple WTO fora. This was thought to potentially make South Africa more vulnerable to non-compliance with newer WTO regulations, exposing them to potential trade-related complaints or disputes. Limited trade literacy within the DoH (outside access to medicines issues) and minimal collaboration and coordination between trade and health policymakers on trade policy development or negotiations was also identified as having potential to reduce nutrition and alcohol policy space.

#### Industry-related characteristics

Industry-related factors include the social acceptability of the industry being regulated with indication that a high level of industry unacceptability can be protective against regulatory chill. For example, in contrast to the food and alcohol industry, trade actors reflected on the social unacceptability of the tobacco industry and its products and how this motivated the government to proceed with plain packaging despite the ongoing recognized risk of a trade or investment dispute.

High levels of social unacceptability of the relevant industry also appeared to diminish the applicability of rationale used by trade actors to explain policy non-decisions. For example, while both trade and health policymakers identified insufficient evidence as a key driver of nutrition policy non-decisions (partly since this exposed South Africa to a trade or investment challenge), lack of evidence of policy effectiveness was not considered a reason to shelve the proposed tobacco plain packaging regulation. As one trade policymaker explained:

“*you can only determine what will be the effect after it has been introduced. So it’s very difficult to anticipate beforehand what the results will be. But from our point of view we don’t really see a negative effect* [of adopting plain packaging].” [DTI1].

A product’s perceived risk to health could also influence the willingness of policymakers to pursue a regulation despite the trade or investment-related legal risks. While strong evidence of a causal relationship between a product and one or more deleterious health outcomes was essential, the health risk of a product appeared also to be assessed on the basis of the complexity of such a causal relationship. For example, one trade policymaker explained the evidence of the risk to health of sugary foods was not considered sufficient to warrant restricting trade.

This was reflected on by one health policy actor as contrasting with South Africa’s willingness to introduce the South African Medicines Act in 1997 despite legal threats that these policies were in violation of TRIPS. This was considered due to how clear and direct the implications of TRIPS was for access to affordable medicines during the AIDS epidemic and the associated scale of AIDS mortality at that time in South Africa. These issue characteristics, along with massive civil society pressure, were cited by policymakers as the key reason government adopted amendments to the Medicines and Related Substance Control Act despite threats of US trade sanctions, a WTO dispute and a domestic legal case brought by multinational pharmaceutical companies including for violations of TRIPs.

#### Cross-border learning

The capacity for cross-border policy learning also appeared to build policymaker confidence in developing regulations that would withstand any trade (or possibly investment) challenge. Health policymakers reported reviewing measures other countries have taken and successfully defended in WTO fora, including the evidence used and policy design. As, for example, one DoH policymaker commented in relation to front-of-pack nutrition labelling:

*“we’re actually looking in terms of what other countries have done and what the challenges might be, we’re involving the legal minds to help come up with something like this, so that we wouldn’t have any trade disputes or any challenges with the WTO”* [DHN1].

Lastly, political will, policy champions and the strength of civil society action were mentioned as important to protect against regulatory chill.

## Discussion

This research sought to investigate if, why and in what form regulatory chill may be occurring in an LMIC country context. Aligned with both previous empirical studies [35, 36], we found a low level of awareness of South Africa’s BIT obligations and the potential threat of an ISDS challenge amongst nutrition and alcohol policymakers and an outsourcing of legal vetting of public health regulations for BIT compliance. While this indicates the potential for investment dispute-related anticipatory chill, we found no definitive evidence of such. However, WTO obligations and the perceived risk of a state-state dispute had contributed to policymakers internalizing a relatively strict evidence-based policymaking approach, general adherence to international standards/guidelines (particularly when local evidence is not available) and a focus on designing regulations to be as least trade restrictive as possible, which together contributed to delayed policy adoption. These findings point to a number of potential strategies to reduce the risk of nutrition and/or alcohol policy chill/non-decisions in South Africa but potentially also other LMICs, although generalizability of our findings are discussed in more detail at the end of this section.

Approaches that may reduce the ‘anticipatory’ burden on health policymaking include, at the international-level, resolving the uncertainty regarding evidential requirements to prove the necessity of a health measure in WTO fora and confirming the acceptability of measures based on existing science or scientific logic in the absence of indisputable evidence of policy effectiveness. Establishing robust mechanisms to manage conflicts of interest within international standard and guideline-setting bodies and fora, including Codex and the WHO will also be critical to reducing industry influence in the standards and guidelines used to shape national policy agendas and protect against trade challenges.

At the national level, increased funding for independent nutrition and alcohol policy research should be a priority. Building capacity within both departments/ministries of health and trade to understand the implications of trade and investment obligations on nutrition and alcohol policy development and establishing new and/or utilizing existing co-ordination mechanisms between departments, to promote health policy expert engagement in trade and investment policy and agreement negotiations will also be important. This may help ensure public health policy space is protected in future agreements, for example by advocating for reducing the burden on health policymakers to prove regulatory effectiveness a priori, instead accepting post-adoption policy evaluation. In South Africa for example a number of mechanisms to promote policy co-ordination across sectors are already well established including the Forum of South African Directors-General cluster system within government and the National Economic Development and Labour Council in which government comes together with business, labour and community groups to discuss and try to reach consensus on issues of social and economic policy. It may be possible to utilize these structures to improve trade and health policy co-ordination. New inter-ministerial co-ordination structures have also recently been established in South Africa which have improved co-ordination on the specific trade and health issue of intellectual property (e.g., the Inter-Ministerial Committee on Intellectual Property). However, structural change alone is insufficient, improving co-ordination and policy cohesion very much depends on each government’s overarching values, interests and priorities in spaces where health issues and wider foreign policy matters converge. Further, co-ordination efforts will only be effective if replicated at the regional (e.g. in SADC model BIT) and international level (e.g. in WTO agreements).

To support such action public health advocacy organizations must become more attuned to the effects of trade agreements on nutrition and alcohol regulatory progress and find ways to distil the complexity of linkages down into simple terms that effectively communicate the implications for the food and alcohol products available in people’s everyday lives their food and alcohol 'environments') [7, 62], which shape food options and drinking decisions. For example, simple messaging of the direct impact of trade agreements on the cost of medicines and people’s health as well as the use of human rights framings proved highly effective in building public support and driving political action to protect access to affordable medicines in South Africa despite threats of trade sanctions, an international trade dispute and domestic litigation.

No clear evidence was identified that THCCs have resorted to threatening South Africa directly with an investor-state dispute in relation to nutrition or alcohol regulations. Rather THCCs tend to seek to protect their status as legitimate stakeholders in policymaking processes and instead use a range of ‘softer’ strategies to influence policy decisions. However, the tobacco plain packaging case provides evidence that by initiating investment litigation against one country, THCCs can generate cross-border response chill, delaying the same regulatory development process in others. This case also suggests that precedential chill may well occur if investment arbitrators rule against a public health regulation. These findings support concerns that a single investor-state dispute can potentially shift decision-making power (at least temporarily) from the state to a private tribunal, not only in the litigating country, but also, in other countries globally [14, 63]. These findings should incentivize LMICs to continue or start taking steps to protect public health policy space within future BITs (e.g. by complete carve-outs of regulations designed to protect public health [64]) and by eliminating their exposure to ISDS, particularly given the limited evidence that investment protection provisions within BITs promote foreign investment [65, 66]. Brazil, for example, has entered into a number of Co-operation and Facilitation Investment Agreements that exclude ISDS [67]. Regionally, consideration of investment protection frameworks that mitigate the risks of earlier investment treaties and establish a more appropriate balance between investor protection and the rights of government to regulate in the public interest may be useful. Given many LMICs’ ongoing exposure to ISDS, increasing public health policymaker knowledge of BIT legal obligations and relevant dispute decisions in a balanced manner such that they can recognize future potential spurious threats and maximize existing policy space, may also be useful.

Trade-related concerns raised by trading partners and industry appear to occur much more frequently than threats of BIT non-compliance in South Africa and have the potential to generate regulatory chill. Down-stream post-treaty adoption strategies to build health policymaker confidence against claims of trade agreement violations may include strengthening mechanisms for policy learning across borders and improved inter-departmental trade and health capacity and coordination, as has been found in Thailand [68]. Alleviating the potentially prohibitive cost for LMICs of defending a health measure in a WTO dispute may also be important to reduce any cost-related drivers of regulatory chill. Requiring public health experts to sit on WTO arbitration panels residing over cases of public health relevance may be a way to decrease the real if not perceived bias of dispute panels.

Diminishing social acceptability of an industry and its product may help shift political priority from avoiding a trade or investment challenge towards a more proactive regulatory approach. Strategies to achieve this include clear communication of a product’s negative impacts on public health; exposure of nefarious industry tactics to promote unhealthy consumption of these products; and use of framing. For example, the industry ‘demonization’ frame has been effective in building public support for regulating the marketing of ‘junk food’ to children in Australia [69] and has widely been applied to promote tobacco control. A ‘systems’ framing for complex public health challenges like obesity that effectively shifts responsibility from the individual to higher level system actors including industry and government has also been effective in generating political priority for obesity prevention in Australia [69].

Finally, careful consideration must be taken before generalizing the key findings described in this single case study to other LMIC settings. Notably, South African trade policymakers may have a heightened awareness of the potential risk of investor-state disputes compared to policymakers in many other LMICs (and especially those engaged in very few trade and/or investment agreements) given South Africa’s previous exposure to investor-state disputes, understanding of the risk to democratic public policymaking posed by the ISDS mechanism (as outlined in its 2010 BIT review), and their ongoing exposure to ISDS under 12 remaining BITs, and under ‘survival’ clauses of terminated agreements. However, while tobacco regulators also recognized this risk, it was not shared by nutrition and alcohol policymakers, which we anticipate may well also be the case in many other countries. As such, findings of very limited investment-dispute related anticipatory chill on nutrition and alcohol regulation in South Africa, may well be similar elsewhere.

We also found South Africa’s previous experience of being threatened with domestic legal action and an international trade dispute over claimed TRIPS violations by its 1997 Medicines Act, may have increased South African policymakers’ level of awareness of trade obligations and dispute risk as compared to health policymakers in LMICs without similar previous experiences. As such, the anticipatory chill of NCD prevention policies to avoid a trade dispute we identified in South Africa may vary in prominence in other LMICs, depending on their trade commitments, previous experience with informal trade challenges or formal WTO disputes and the specific health policy area.

Direct threats of an investment or trade dispute challenging NCD prevention policies in LMICs with even more limited financial, legal and administrative resources and/or lower prioritization of NCDs and weaker public health policy norms than South Africa, may make other LMICs more vulnerable to response chill than we found in our case study.

Overall however, despite the variable degrees of regulatory chill likely to be occurring in different countries and in different NCD policy areas, we suggest that the ongoing and deepening commitment to trade and investment obligations in many countries, and the potential for corporations to use these to threaten costly trade or investment disputes, make the recommendations in this paper widely applicable.

### Limitations

In addition to generalizability considerations, there are two other key limitations in our analysis. Firstly, we interviewed significantly fewer higher-level trade and health political actors than those leading policy development at the technical level. This may have meant certain relevant high-level negotiations, deal-brokering or cases of trade or investment dispute-related regulatory chill were not captured in this analysis. Secondly, the analysis may be limited due to nondisclosure of relevant information by interviewed stakeholders due to the powerful interests involved and political nature of the topics covered.

## Conclusion

To the best of our knowledge this research contributes the first case study investigating trade and investment dispute-related public health regulatory chill in an LMIC country context. Our findings indicate that, at the time of our study, South Africa’s trade obligations had a more prominent role in nutrition and alcohol regulatory chill than BIT-related concerns. However, given the potential for wider use of the ISDS mechanism by THCCs in the future, strategies to protect public health policy space in the context of both international trade and investment treaty and dispute settlement contexts will be important. This work highlights the need for further research examining strategies used by governments to withstand BIT and trade-related legal threats by industry (or trading partners) and how greater protection of health policy space can be achieved within trade and investment agreements.

## Data Availability

The data that support the findings of this study are available, with some restrictions applied, on reasonable request from the corresponding author [PM]. The data are not publicly available due to them containing information that could compromise research participant privacy/consent.
